# Comparative Analysis of Gastrointestinal Morphology and Enteric Nervous System Organization in Mallard, Tufted Duck, and Green-Winged Teal

**DOI:** 10.3390/ani15172511

**Published:** 2025-08-26

**Authors:** Ligia Janicka, Aleksandra Dajnowska, Cezary Osiak-Wicha, Katarzyna Kras, Marian Flis, Katarzyna Woźniak, Marcin B. Arciszewski

**Affiliations:** 1 Department of Animal Anatomy and Histology, Faculty of Veterinary Medicine, University of Life Sciences in Lublin, Akademicka 12, 20-950 Lublin, Poland; aleksandra.dajnowska@up.lublin.pl (A.D.); cezary.wicha@up.lublin.pl (C.O.-W.); katarzyna.kras@up.lublin.pl (K.K.); katarzyna.wozniak@up.lublin.pl (K.W.); mb.arciszewski@wp.pl (M.B.A.); 2 Department of Ethology and Wildlife Management, Faculty of Animal Sciences and Bioeconomy, University of Life Sciences in Lublin, Akademicka 13, 20-950 Lublin, Poland; marian.flis@up.lublin.pl

**Keywords:** ducks, gastrointestinal morphology, enteric nervous system (ENS), intestinal villi, intestinal crypts, trophic adaptation, morphometry, waterfowl

## Abstract

Ducks exhibit substantial ecological and dietary diversity, which drives morphological and functional adaptations in their digestive systems. This study analyzed the small intestine and cecum of three wild duck species: Mallard (*Anas platyrhynchos*), Tufted Duck (*Aythya fuligula*), and Green-Winged Teal (*Anas crecca*) collected post-mortem. Histomorphometric analysis and immunohistochemistry (IHC) with the pan-neuronal marker HuC/D were performed. The Tufted Duck showed the thickest intestinal muscle layers, particularly in the duodenum and ileum, and the largest enteric ganglia, indicating adaptation to a fibrous and protein-rich diet. The Mallard displayed the longest villi and deepest crypts, consistent with its omnivorous diet rich in plant material. The Green-Winged Teal, which consumes highly digestible insect-rich food, had the shortest villi and thinnest muscle layers. Differences in enteric ganglion size and organization among species suggest varying neuroregulatory demands in different gut segments. These findings confirm that intestinal morphology and enteric nervous system (ENS) structure are tightly linked to dietary specialization and ecological strategies. The results highlight the high adaptive plasticity of the avian digestive system in response to feeding behavior.

## Simple Summary

This study examined the intestine morphology and enteric nervous systems (ENSs) of three duck species with different feeding strategies: the Mallard, Tufted Duck, and Green-Winged Teal. The differences in their diets were reflected in the structure of their intestines and ENSs. The Tufted Duck, which consumes hard aquatic food, had the thickest intestinal muscles and the largest enteric ganglia. The Mallard, with an omnivorous diet rich in plant material, showed the longest intestinal villi and deepest crypts. The Green-Winged Teal, feeding on soft, insect-rich food, had thinner intestinal walls and shorter villi. These results highlight how the structure of the gastrointestinal tract (GIT) and ENS is closely related to the type of diet and feeding habits of each species.

## 1. Introduction

Ducks (*Anatinae*) are a diverse and ecologically significant group of waterfowl, characterized by their adaptability, migratory behaviors, and their critical role in ecosystem dynamics [1]. Among the species that inhabit wetlands, lakes, rivers, and coastal regions, three stand out due to their distinctive ecological adaptations: the Mallard (*Anas platyrhynchos*), Tufted Duck (*Aythya fuligula*), and Green-Winged Teal (*Anas crecca*) [2,3]. These species represent variations in habitat preferences, diet, and behavior, which may lead to physiological differences, particularly in the gastrointestinal tract (GIT) and enteric nervous system (ENS), the focus of this study.

The Mallard is a highly adaptable species, often found in a wide range of aquatic environments, including urban areas. This omnivorous bird feeds on a variety of plant matter, small invertebrates, and even small fish, reflecting its ability to exploit diverse habitats [4,5]. The Tufted Duck, on the other hand, is more specialized in its feeding behavior, primarily consuming aquatic vegetation, mollusks, and invertebrates from deeper waters due to its proficient diving ability [6]. The Green-Winged Teal, the smallest of the three species, inhabits shallow wetlands and marshes and exhibits a more insectivorous diet, feeding largely on small aquatic invertebrates and seeds [4]. These differences in diet and habitat between the species suggest potential morphological and neural adaptations in their GITs, which play a critical role in digestion, nutrient absorption, and overall survival [7,8].

The gastrointestinal morphology of birds, particularly the small intestine, varies considerably between species, reflecting adaptations to their dietary preferences [7,9]. Morphological features such as villi size and density, mucosal thickness, and the overall structure of the intestinal layers are directly influenced by diet and habitat [10,11]. For instance, birds with diets rich in fibrous plant matter typically exhibit longer intestines and more developed villi, enhancing their ability to absorb nutrients from complex carbohydrates, whereas carnivorous or insectivorous birds may have shorter intestines optimized for rapid nutrient absorption from protein-rich diets [12,13]. Investigating these morphological differences in the small intestines of the Mallard, Tufted Duck, and Green-Winged Teal can offer insights into how their digestive systems have adapted to their ecological niches.

In addition to the morphological differences, the ENS is a critical but often overlooked aspect of gastrointestinal function. In birds, the ENS is organized into two main plexuses: the myenteric plexus, located between the layers of the muscularis externa, and the submucosal plexus, situated in the submucosa. These plexuses contain various types of neurons, including cells expressing the Hu C/D protein—a neuronal marker used in this study to assess the density and distribution of enteric neurons. The presence and variation in these neurons can provide insights into how these ducks’ nervous systems have adapted to manage the physiological demands of their specific diets and habitats [14].

Given the Mallard’s broad dietary adaptability, the Tufted Duck’s specialized diving behavior, and the Green-Winged Teal’s insectivorous diet, we hypothesize that there will be significant differences in both the morphological characteristics of the small intestine and the organization of the ENS among these species. Specifically, we expect the Tufted Duck to exhibit more robust intestinal muscles and larger enteric ganglia as an adaptation to diet rich in hard, structurally resistant prey. However, differences in ENS organization between species are likely influenced by multiple factors. While muscle layer thickness may affect the size, distribution, and density of ganglia, other elements such as intestinal motility patterns, functional demands related to specific diets, and species-specific ecological adaptations may also contribute. Considering these aspects allows for a broader understanding of how gastrointestinal morphology and ENS structure co-evolve to meet the physiological needs of each species. The Mallard, as an omnivorous dabbling species with a mixed diet of aquatic plants and invertebrates, is anticipated to exhibit intermediate gastrointestinal characteristics, while the Green-Winged Teal may display a shorter, more streamlined small intestine suited to its insectivorous diet. Additionally, the ENS of each species may show variations in neuron density and distribution, particularly in the myenteric and submucosal plexuses, reflecting their different digestive demands.

## 2. Materials and Methods

### 2.1. Animals and Material

The study material consisted of deceased individuals from three duck species: Tufted Duck (*Aythya fuligula*), Mallard (*Anas platyrhynchos*), and Green-Winged Teal (*Anas crecca*), obtained during the legal hunting season in July 2023 in accordance with the Polish Hunting Law Act of 13 October 1995 (Journal of Laws 1995 No. 147, item 713; https://isap.sejm.gov.pl/isap.nsf/DocDetails.xsp?id=wdu19951470713; accessed on 26 July 2004). Ethical approval was not required for this research, as all samples were collected post-mortem. The focus of the study was the digestive tract, specifically the small intestine (duodenum, jejunum, and ileum) and the cecum. A total of 18 individuals—six from each species—were examined. The birds were randomly selected regardless of sex.

### 2.2. Tissue Samples Collection

All individuals were adults, as determined by body size and plumage characteristics, and tissue samples were fixed as soon as possible post-mortem (within 2 h). Following anatomical dissection, segments of the small intestine (duodenum, jejunum, and ileum) and the cecum were collected and carefully cleaned of intestinal contents and surrounding tissues using surgical scissors and scalpels, with approximately 30 mm taken from each section. The samples were fixed in 4% buffered formaldehyde (pH 7.0) for 24 h and then thoroughly rinsed under running tap water. Tissues were dehydrated in graded ethanol concentrations and subsequently cleared in xylene to prepare them for paraffin embedding. The embedding process was carried out using a modular embedding station (MYR EC-350, Casa Álvarez Material Científico SA, Madrid, Spain). Paraffin blocks were sectioned into 5 µm-thick slices using a rotary microtome (HM 360, Microm, Walldorf, Germany). Sections were mounted on SuperFrost^®^ Plus microscope slides (Thermo Fisher Scientific, Menzel-Gläser, Braunschweig, Germany) and dried on a heating plate at 48 °C.

### 2.3. Histomorphometrical Analysis

Goldner’s trichrome staining was used to visualize the intestinal architecture and differentiate the histological layers of the intestinal wall [15]. The specimens were examined using a BX-51 DSU light microscope (Olympus, Tokyo, Japan) equipped with a DP-70 digital camera (Olympus, Tokyo, Japan) at magnifications of 100× and 200×. High-resolution images were captured by a single operator using Cell^M v2.3 (Olympus, Tokyo, Japan) and cellSens Standard software (v1.18, Build 18686, Olympus, Tokyo, Japan) under consistent lighting conditions and uniform brightness and contrast settings.

**Figure 1:**
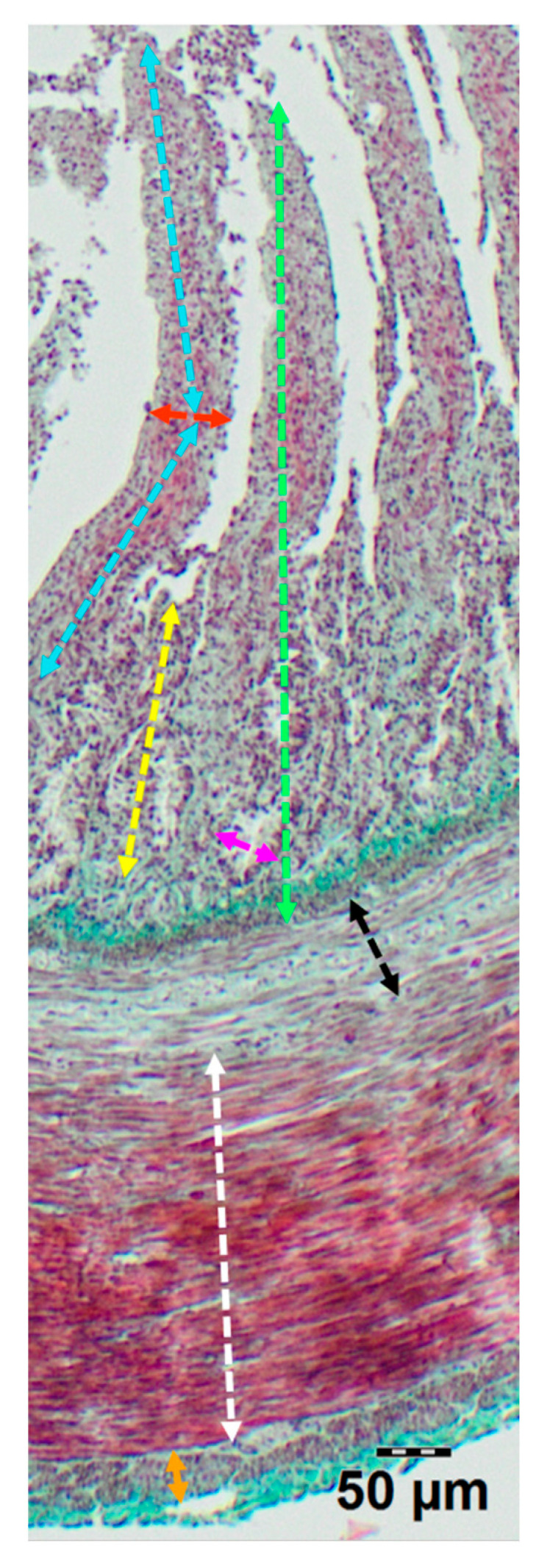
Representative image of duodenum wall showing measurement scheme of morphological parameters: villi height (light blue line), villi width (red line), mucosa thickness (green line), submucosa thickness (black line), crypt depth (yellow line), crypt width (pink line), thickness of the longitudinal muscle layer (orange line), thickness of the circular muscle layer (white line).

Morphometric evaluation included measurements of the thickness of the inner and outer muscular layers, as well as the mucosa and submucosa. Additionally, the depth of the intestinal crypts (measured from the base of the crypt to the base of the adjacent villus) and their width (measured at the midpoint of the crypt depth) were assessed. The height of the villi (from the tip to the villus-crypt junction) and their width (measured at the mid-height) were also determined (Figure 1). Furthermore, the total number of crypts and the number of villi per 1 mm of the mucosal surface were quantified in cross sections by counting them along a measured 1 mm segment of the mucosa. Only well-preserved villi and crypts, sectioned perpendicularly to the mucosal surface, were included in the morphometric analysis. Damaged or obliquely cut structures were excluded from measurements. For each animal, measurements were taken from several randomly selected microscopic fields and averaged. All measurements were performed twice by the same observer to minimize intra-observer variability. The orientation of all sections was standardized to ensure that villi and crypts were cut perpendicular to the mucosal surface.

### 2.4. Immunohistochemistry (IHC) Analysis

Immunohistochemical staining was performed using mouse monoclonal primary antibodies against HU (human neuronal protein HuC/D—a pan-neuronal marker). The antibodies used in the study are described in (Table 1). Tissue sections were deparaffinized in xylene (3× for 5 min), rehydrated through a graded series of ethanol solutions (100–50%), and rinsed in deionized water. Endogenous peroxidase activity was blocked using 3% hydrogen peroxide for 10 min at room temperature. Antigen retrieval was carried out in citrate buffer (pH 6.0) using a pressure cooker (8 min at 80 °C, multicooker RMC-PM381-E, Redmond Industrial Group, Moscow, Russia). After rinsing in Phosphate-Buffered Saline (PBS), a protein blocking serum was applied for 5 min (UltraVision Protein Block; Thermo Fisher Scientific, Waltham, MA, USA), followed by overnight incubation with the primary antibody at 4 °C in a humidified chamber. On the following day, a two-step detection system was used with a goat polyclonal Horseradish Peroxidase (HRP)-conjugated secondary antibody (ImmunoLogic WellMed B.V., Duiven, The Netherlands, Table 1), applied for 30 min at room temperature. Negative controls were performed by replacing the primary antibody with PBS, which resulted in the absence of specific staining. Immunoreactivity was visualized using 3,3′-diaminobenzidine (DAB substrate kit; ab64238; Abcam, Cambridge, UK), and the sections were counterstained with Mayer’s hematoxylin (Patho, Mar-Four, Konstantynów Łódzki, Poland), dehydrated through a graded ethanol series (50–100%), cleared in xylene, and mounted under coverslips. Microscopic examination was performed using a light microscope (BX-51 DSU, Olympus, Tokyo, Japan) equipped with a digital color camera at magnifications of 100× and 200×.

In addition, a morphometric analysis of neuronal ganglia immunoreactive to the neuronal protein HuC/D was performed. This included measurements of ganglion length, width, and surface area. All measurements were carried out on the same intestinal sections using calibrated microscopic images. For each intestinal segment, ganglia were assessed in multiple non-adjacent cross sections obtained at regular intervals throughout the paraffin block to minimize the risk of missing structures due to uneven distribution or sectioning plane. Measurements were taken only from well-preserved ganglia with clearly defined borders in the myenteric plexus.

### 2.5. Statistical Analysis

All statistical analyses were conducted using GraphPad Prism version 10.5.0 for Windows (GraphPad Software, San Diego, CA, USA). Prior to inferential testing, data distributions were assessed for normality using the Shapiro–Wilk test, and Levene’s test was employed to evaluate the homogeneity of variances. For normally distributed data with equal variances, a one-way analysis of variance (ANOVA) was performed to compare parameters of intestinal morphology and the ENS among the three duck species. When significant differences were detected (*p* < 0.05), Tukey’s post hoc test was used for pairwise comparisons. For non-normally distributed data, the Kruskal–Wallis test was used, with Dunn’s multiple comparison test applied post hoc where appropriate. All data are presented as mean ± standard error of the mean (SEM), and the level of statistical significance was set at *p* < 0.05.

## 3. Results

**Figure 2:**
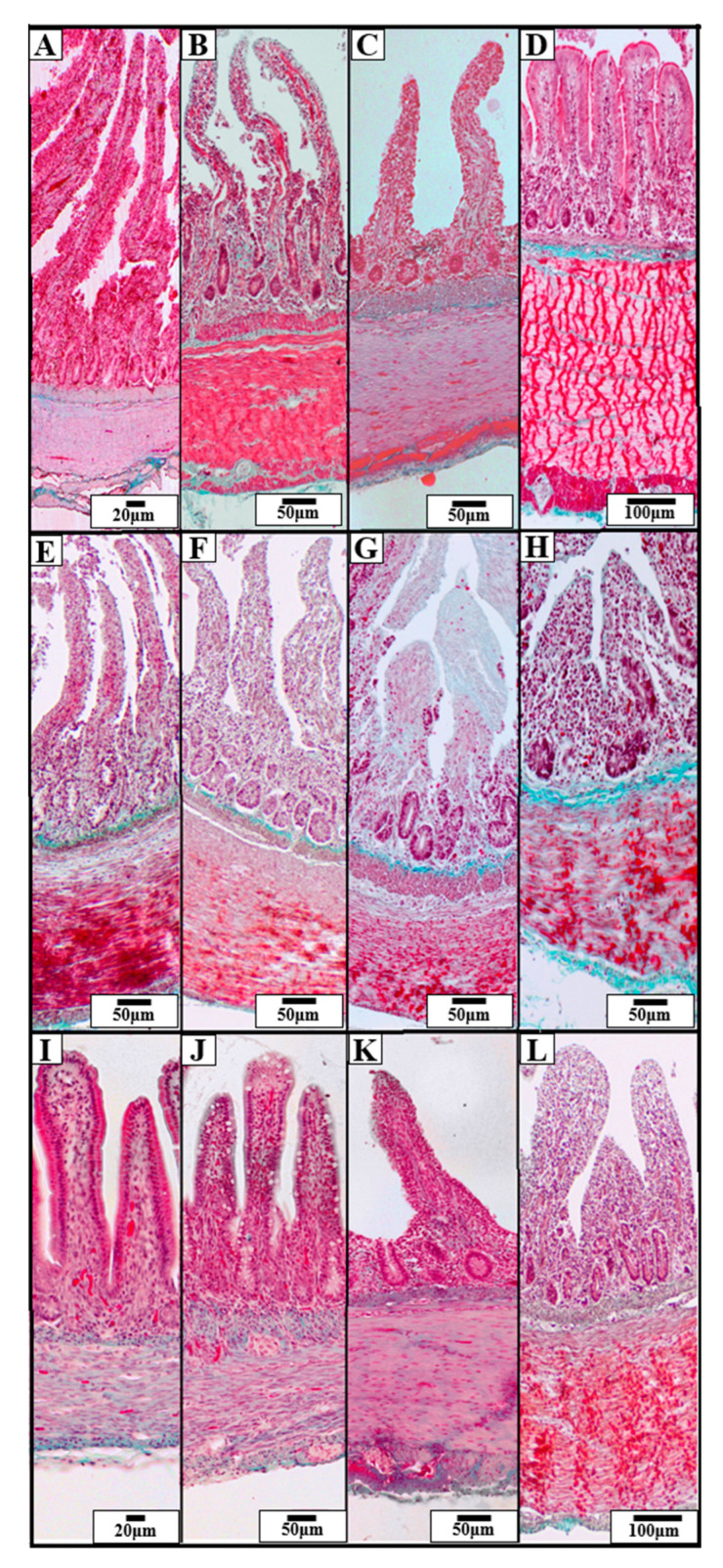
Representative photomicrographs of Goldner’s staining showing the structure of the intestinal wall in three species of wild ducks: Mallard (*Anas platyrhynchos*) (**A**–**D**), Tufted Duck (*Aythya fuligula*) (**E**–**H**), and Green-Winged Teal (*Anas crecca*) (**I**–**L**). The visible sections of the GIT include: duodenum (**A**,**E**,**I**), jejunum (**B**,**F**,**J**), ileum (**C**,**G**,**K**), and cecum (**D**,**H**,**L**). The images show morphological differences in the villi, crypts, and muscular and submucosal layers. Scale bars are shown in each image: 20 µm, 50 µm, 100 µm.

A comparative analysis among the three duck species—Mallard, Tufted Duck, and Green-Winged Teal—revealed significant differences in the morphometric structure of individual intestinal segments (Figure 2).

### 3.1. Duodenum Morphometry

**Figure 3:**
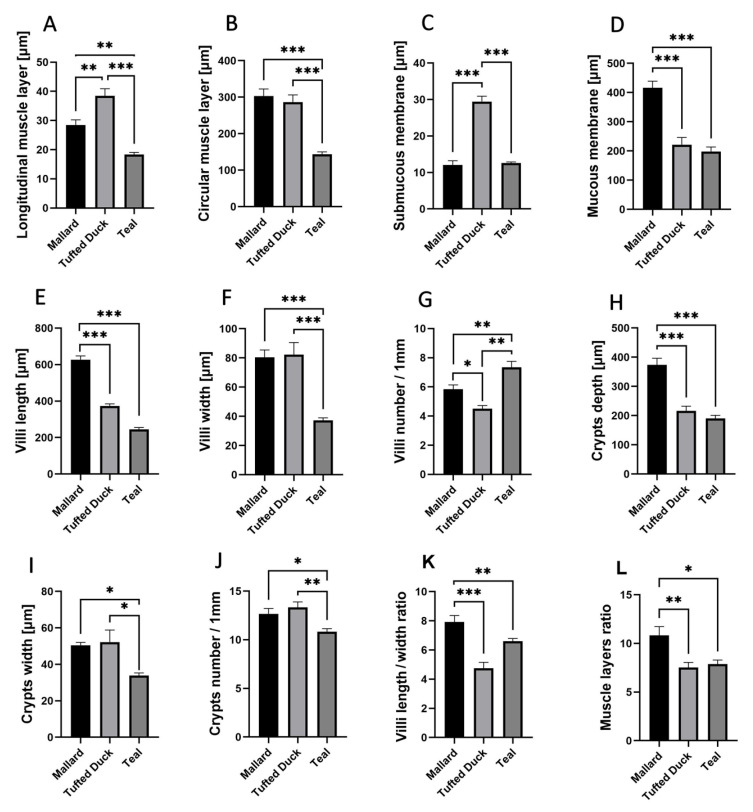
Morphometric characteristics of the duodenum in Mallard, Tufted Duck, and Green-Winged Teal. (**A**) Thickness of the longitudinal muscle layer, (**B**) Thickness of the circular muscle layer, (**C**) Thickness of the submucosa, (**D**) Thickness of the mucosa, (**E**) Length of villi, (**F**) Width of villi, (**G**) Number of villi per 1 mm of mucosa, (**H**) Depth of intestinal crypts, (**I**) Width of intestinal crypts, (**J**) Number of crypts per 1 mm of mucosa, (**K**) Villi length/width ratio, (**L**) Muscle layers (longitudinal to circular muscle layer thickness) ratio. Data are presented as mean ± SEM. * *p* < 0.05, ** *p* < 0.01, *** *p* < 0.001; *n* = 6 per species.

The longitudinal muscle layer (Figure 3A) was significantly thicker in the Tufted Duck compared to the Mallard (*p* < 0.01) and Green-Winged Teal (*p* < 0.001). A similar pattern was observed in the circular muscle layer (Figure 3B), where the Green-Winged Teal differed significantly from the Mallard and Tufted Duck (*p* < 0.001) for both comparisons. The thickness of the submucosa (Figure 3C) was greatest in the Tufted Duck compared to the other species (*p* < 0.001), while the thickness of the mucosa (Figure 3D) was greatest in the Mallard (*p* < 0.001). Villus length (Figure 3E) was highest in the Mallard and significantly greater than in the Tufted Duck and Green-Winged Teal (*p* < 0.001). The width of the villi (Figure 3F) was significantly greater in the Tufted Duck and the Mallard compared to the Green-Winged Teal (*p* < 0.001). The number of villi per 1 mm of mucosa (Figure 3G) was highest in the Green-Winged Teal (*p* < 0.01), with significant differences compared to the Tufted Duck and the Mallard. Additionally, the number of villi was significantly lower in the Tufted Duck than in the Mallard (*p* < 0.05). Crypt depth (Figure 3H) was significantly greater in the Mallard than in the Green-Winged Teal and the Tufted Duck (*p* < 0.001). The width of the intestinal crypts (Figure 3I) was noticeably smaller in the Green-Winged Teal compared to both the Mallard and the Tufted Duck (*p* < 0.05). Similarly, the number of crypts per 1 mm of mucosa (Figure 3J) was also the lowest in the Green-Winged Teal, with significant differences observed in comparison to the Mallard (*p* < 0.05) and the Tufted Duck (*p* < 0.01). The villus length-to-width ratio (Figure 3K) was significantly greater in the Mallard compared to the Tufted Duck (*p* < 0.001) and the Green-Winged Teal (*p* < 0.01). No significant differences were observed between the Tufted Duck and the Green-Winged Teal. The muscle layer thickness ratio (Figure 3L) was significantly greater in the Mallard compared to the Tufted Duck (*p* < 0.01) and the Green-Winged Teal (*p* < 0.05). No significant differences were observed between the Tufted Duck and the Green-Winged Teal.

### 3.2. Jejunum Morphometry

**Figure 4:**
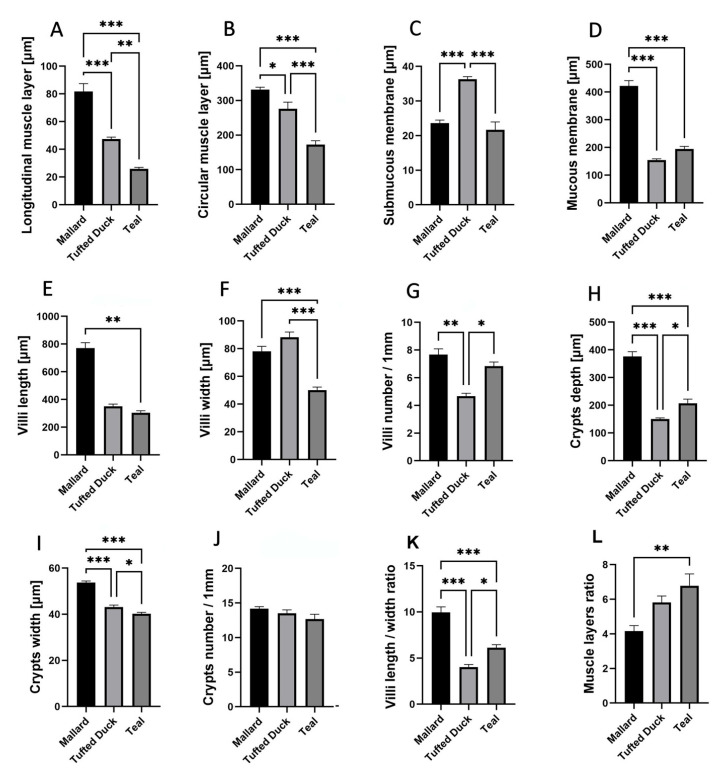
Morphometric characteristics of the jejunum in Mallard, Tufted Duck, and Green-Winged Teal. (**A**) Thickness of the longitudinal muscle layer, (**B**) Thickness of the circular muscle layer, (**C**) Thickness of the submucosa, (**D**) Thickness of the mucosa, (**E**) Length of villi, (**F**) Width of villi, (**G**) Number of villi per 1 mm of mucosa, (**H**) Depth of intestinal crypts, (**I**) Width of intestinal crypts, (**J**) Number of crypts per 1 mm of mucosa, (**K**) Villi length/width ratio, (**L**) Muscle layers (longitudinal to circular muscle layer thickness) ratio. Data are presented as mean ± SEM. * *p* < 0.05, ** *p* < 0.01, *** *p* < 0.001; *n* = 6 per species.

In the jejunum, the thickness of the longitudinal muscle layer (Figure 4A) was significantly greater in the Mallard compared to both the Tufted Duck and the Green-Winged Teal (*p* < 0.001). Additionally, the Tufted Duck exhibited significantly greater thickness than the Green-Winged Teal (*p* < 0.01). The circular muscle layer (Figure 4B) was thinnest in the Green-Winged Teal, with significant differences compared to the other two species (*p* < 0.001), while the Mallard showed significantly greater thickness than the Tufted Duck (*p* < 0.05). The submucosa (Figure 4C) was thickest in the Tufted Duck, with highly significant differences compared to both the Mallard and the Green-Winged Teal (*p* < 0.001). The mucosa (Figure 4D) was significantly thicker in the Mallard compared to the Tufted Duck and the Green-Winged Teal (*p* < 0.001). Villus length (Figure 4E) was significantly greater in the Mallard compared to the Green-Winged Teal (*p* < 0.01). Villus width (Figure 4F) was smallest in the Green-Winged Teal, with highly significant differences compared to both the Mallard and the Tufted Duck (*p* < 0.001). The number of villi per 1 mm of mucosa (Figure 4G) was lowest in the Tufted Duck, with statistically significant differences compared to the Mallard (*p* < 0.01) and the Green-Winged Teal (*p* < 0.05). Crypt depth (Figure 4H) was significantly greater in the Mallard compared to the other species (*p* < 0.01), while the Green-Winged Teal showed significantly greater depth than the Tufted Duck (*p* < 0.05). Crypt width (Figure 4I) was also greatest in the Mallard (*p* < 0.001), and the Tufted Duck had significantly wider crypts than the Green-Winged Teal (*p* < 0.05). No significant differences were observed in the number of crypts per 1 mm of mucosa among the species (Figure 4J). The villus length-to-width ratio (Figure 4K) was significantly highest in the Mallard compared to the Tufted Duck (*p* < 0.001) and the Green-Winged Teal (*p* < 0.01). Furthermore, the Green-Winged Teal had a significantly higher value of this parameter compared to the Tufted Duck (*p* < 0.05). The muscle layer thickness ratio (Figure 4L) was significantly greater in the Green-Winged Teal compared to the Mallard (*p* < 0.01). No significant differences were found between the Tufted Duck and the Green-Winged Teal or between the Tufted Duck and the Mallard.

### 3.3. Ileum Morphometry

**Figure 5:**
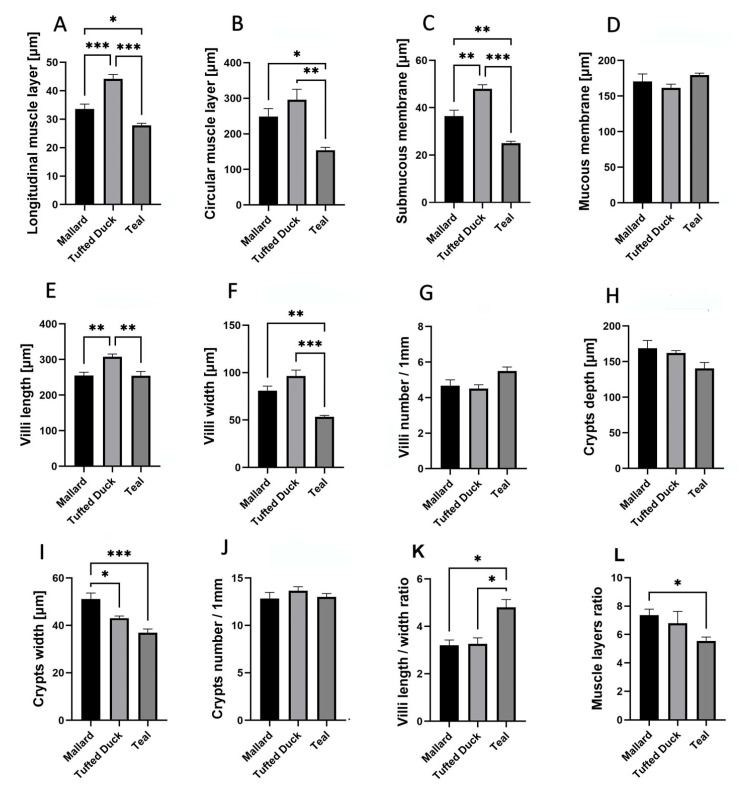
Morphometric characteristics of the ileum in Mallard, Tufted Duck, and Green-Winged Teal. (**A**) Thickness of the longitudinal muscle layer, (**B**) Thickness of the circular muscle layer, (**C**) Thickness of the submucosa, (**D**) Thickness of the mucosa, (**E**) Length of villi, (**F**) Width of villi, (**G**) Number of villi per 1 mm of mucosa, (**H**) Depth of intestinal crypts, (**I**) Width of intestinal crypts, (**J**) Number of crypts per 1 mm of mucosa, (**K**) Villi length/width ratio, (**L**) Muscle layers (longitudinal to circular muscle layer thickness) ratio. Data are presented as mean ± SEM. * *p* < 0.05, ** *p* < 0.01, *** *p* < 0.001; *n* = 6 per species.

In the ileum, the thickness of the longitudinal muscle layer (Figure 5A) was significantly greater in the Tufted Duck compared to both the Mallard and the Green-Winged Teal (*p* < 0.001). Additionally, the Mallard showed a noticeably greater thickness than the Green-Winged Teal (*p* < 0.05). The circular muscle layer (Figure 5B) was thinnest in the Green-Winged Teal, with statistically significant differences compared to the Tufted Duck (*p* < 0.01) and the Mallard (*p* < 0.05). The submucosa (Figure 5C) was thickest in the Tufted Duck, with significant differences relative to the Mallard (*p* < 0.01) and the Green-Winged Teal (*p* < 0.001). Furthermore, the submucosa was significantly thicker in the Mallard than in the Green-Winged Teal (*p* < 0.01). Villus length (Figure 5E) was significantly greater in the Tufted Duck compared to both the Mallard and the Green-Winged Teal (*p* < 0.01). Villus width (Figure 5F) was smallest in the Green-Winged Teal, showing significant differences compared to the Mallard (*p* < 0.01) and the Tufted Duck (*p* < 0.001), while no significant difference was observed between the Mallard and the Tufted Duck. Crypt width (Figure 5I) was significantly greater in the Mallard compared to the Tufted Duck (*p* < 0.05) and the Green-Winged Teal (*p* < 0.001). No statistically significant differences among the species were observed in mucosal thickness (Figure 5D), the number of villi per 1 mm of mucosa (Figure 5G), crypt depth (Figure 5H), or the number of crypts per 1 mm (Figure 5J). The villus length-to-width ratio (Figure 5K) was significantly highest in the Green-Winged Teal compared to the other species (*p* < 0.05). No significant differences were observed between the Mallard and the Tufted Duck. The muscle layer thickness ratio (Figure 5L) was significantly greater in the Mallard compared to the Green-Winged Teal (*p* < 0.05). No significant differences were found between the Mallard and the Tufted Duck or between the Tufted Duck and the Green-Winged Teal.

### 3.4. Cecum Morphometry

**Figure 6:**
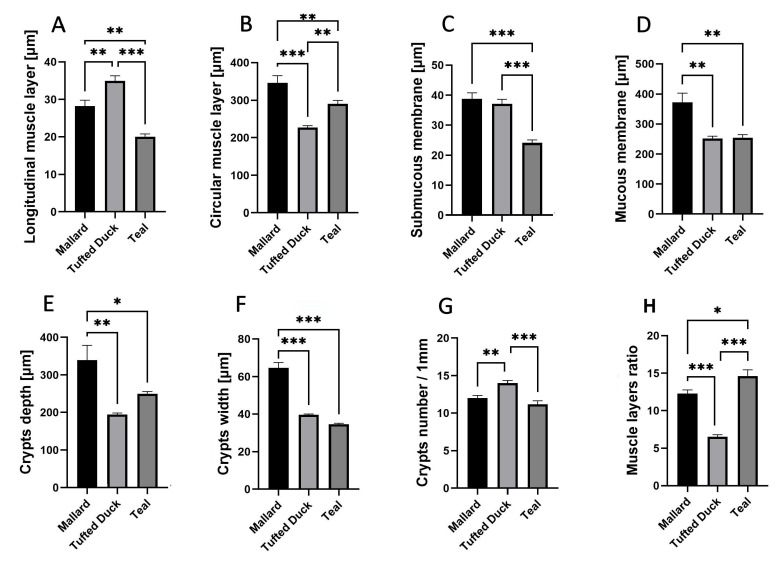
Morphometric characteristics of the cecum in Mallard, Tufted Duck, and Green-Winged Teal. (**A**) Thickness of the longitudinal muscle layer, (**B**) Thickness of the circular muscle layer, (**C**) Thickness of the submucosa, (**D**) Thickness of the mucosa (**E**) Depth of intestinal crypts, (**F**) Width of intestinal crypts, (**G**) Number of crypts per 1 mm of mucosa, (**H**) Muscle layers (longitudinal to circular muscle layer thickness) ratio. Data are presented as mean ± SEM. * *p* < 0.05, ** *p* < 0.01, *** *p* < 0.001; *n* = 6 per species.

In the cecum, the Tufted Duck exhibited the greatest thickness of the longitudinal muscle layer (Figure 6A) compared to the Green-Winged Teal (*p* < 0.001) and the Mallard (*p* < 0.01). Additionally, the Mallard showed significantly greater thickness than the Green-Winged Teal (*p* < 0.01). The circular muscle layer (Figure 6B) was thickest in the Mallard, with significant differences compared to the Tufted Duck (*p* < 0.001) and the Green-Winged Teal (*p* < 0.01). At the same time, the Green-Winged Teal showed significantly greater thickness than the Tufted Duck (*p* < 0.01). The thickness of the submucosa (Figure 6C) was lowest in the Green-Winged Teal compared to the other species (*p* < 0.001), while no significant differences were observed between the Tufted Duck and the Mallard. The mucosa (Figure 6D) was thickest in the Mallard, with statistically significant differences compared to both the Tufted Duck and the Green-Winged Teal (*p* < 0.01); however, no significant differences were found between the Tufted Duck and the Green-Winged Teal. Crypt depth (Figure 6E) was greatest in the Mallard, with significant differences compared to the Tufted Duck (*p* < 0.01) and the Green-Winged Teal (*p* < 0.05). Crypt width (Figure 6F) was also greatest in the Mallard compared to both other species (*p* < 0.001); no significant differences were observed between the Tufted Duck and the Green-Winged Teal. The number of crypts per 1 mm of mucosa (Figure 6G) was highest in the Tufted Duck, with significant differences compared to the Mallard (*p* < 0.01) and the Green-Winged Teal (*p* < 0.001). The muscle layer thickness ratio (Figure 6H) was significantly greater in the Green-Winged Teal compared to the Tufted Duck (*p* < 0.001) and the Mallard (*p* < 0.05). Furthermore, the Mallard had a significantly higher value of this parameter compared to the Tufted Duck (*p* < 0.001).

### 3.5. Morphometry of Enteric Ganglia

**Figure 7:**
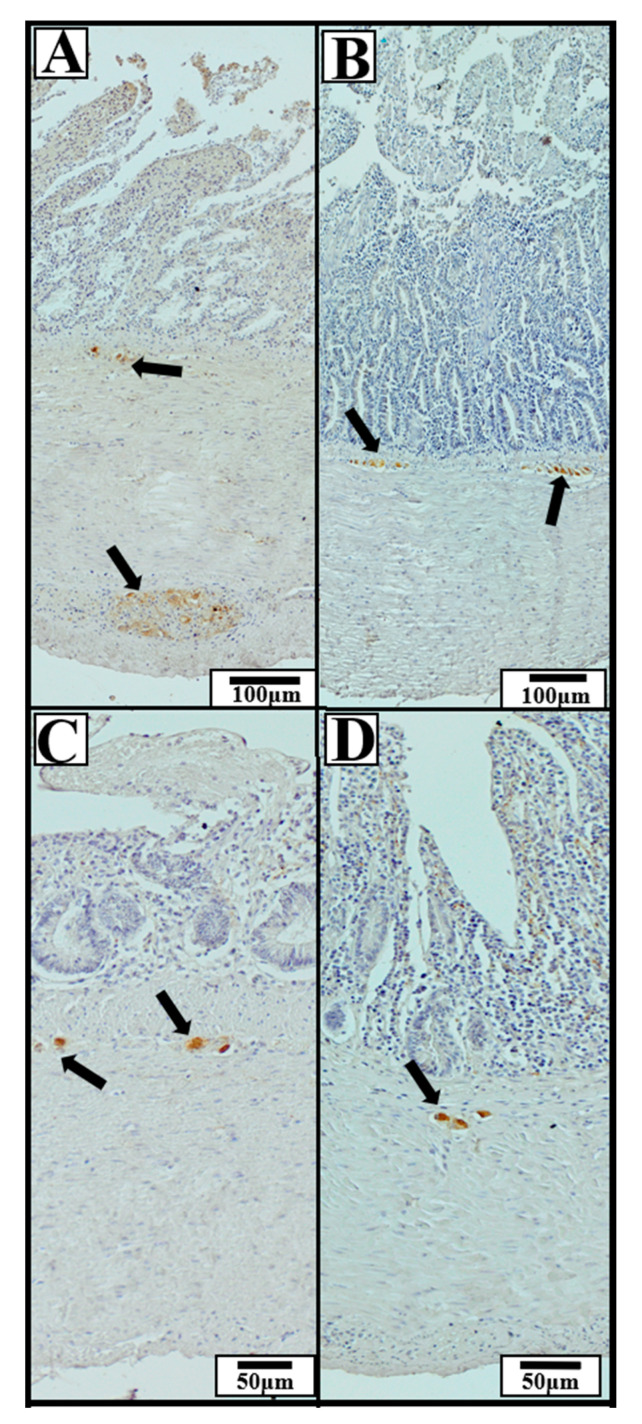
Representative immunohistochemical photomicrographs of the small and cecal intestinal wall in wild ducks, showing HuC/D-immunoreactive neuronal ganglia (neuronal marker). Black arrows indicate ganglia exhibiting positive immunohistochemical staining. The presented sections of the GIT include: (**A**) duodenum, (**B**) jejunum, (**C**) ileum, (**D**) cecum. The images illustrate typical localization and morphology of ganglia within specific intestinal segments, regardless of species. Scale bars: 50 µm and 100 µm.

Significant interspecies differences were observed in the dimensions of HuC/D-immunoreactive ganglia in selected intestinal segments (Figure 7).

**Figure 8:**
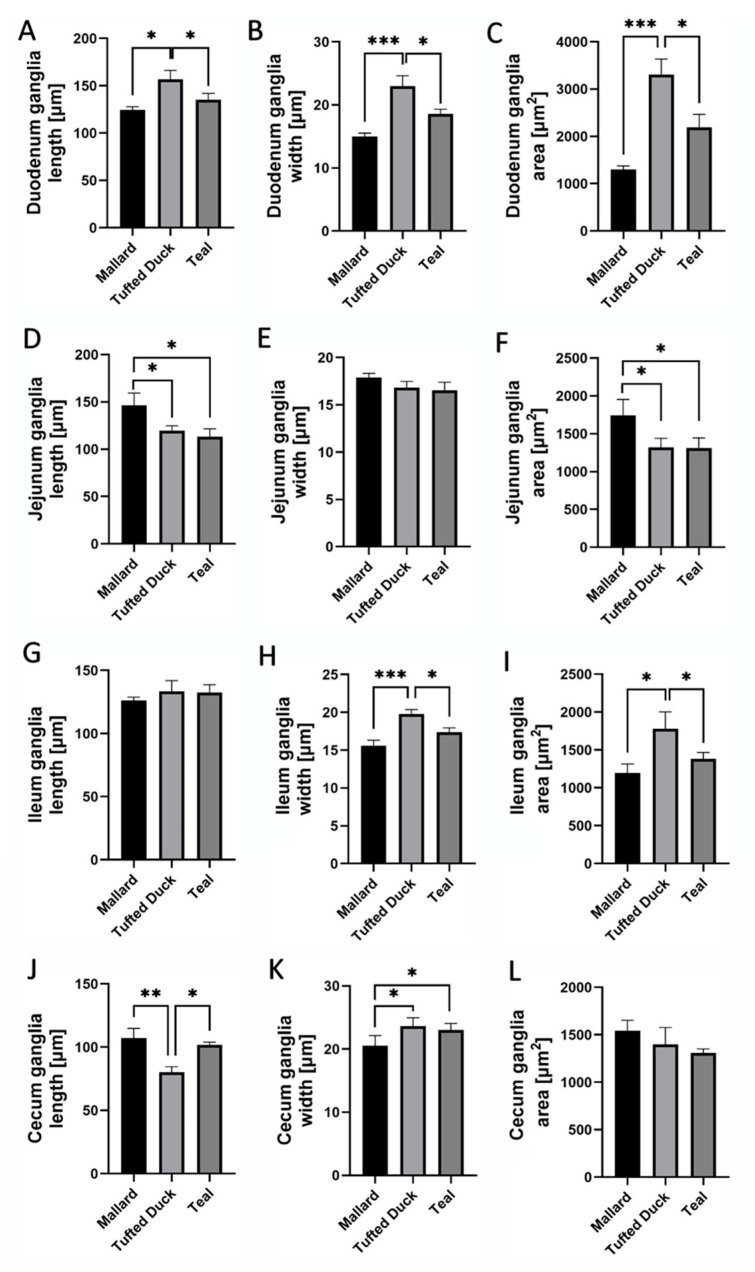
Morphometric characteristics of HuC/D-immunoreactive ganglia in the duodenum, jejunum, ileum, and cecum of Mallard, Tufted Duck, and Green-Winged Teal. (**A**–**C**) Ganglia length, width, and area in the duodenum, (**D**–**F**) Ganglia length, width, and area in the jejunum, (**G**–**I**) Ganglia length, width, and area in the ileum, (**J**–**L**) Ganglia length, width, and area in the cecum. Data are expressed as mean ± SEM. Asterisks indicate significant differences between groups: * *p* < 0.05, ** *p* < 0.01, *** *p* < 0.001; *n* = 6 per species.

In the duodenum, the Tufted Duck exhibited the highest values of ganglion length, width, and area compared to the other examined species. Ganglia length was significantly greater in the Tufted Duck than in both the Green-Winged Teal and the Mallard (*p* < 0.05), with no significant differences observed between the Mallard and the Green-Winged Teal (Figure 8A). Ganglia width was also significantly greater in the Tufted Duck compared to the Mallard (*p <* 0.001) and the Green-Winged Teal (*p* < 0.05), while no significant differences were found between the Mallard and the Green-Winged Teal (Figure 8B). Similarly, ganglia area was significantly larger in the Tufted Duck than in the Mallard (*p* < 0.001) and the Green-Winged Teal (*p* < 0.05), with no significant differences between the latter two species (Figure 8C).

In the jejunum, ganglia length was significantly greater in the Mallard than in the Tufted Duck and Green-Winged Teal (*p* < 0.05) (Figure 8D). There were no significant differences in ganglia width among the species (Figure 8E). However, the ganglia area was significantly higher in the Mallard compared to the Tufted Duck and Green-Winged Teal (*p* < 0.05) (Figure 8F).

In the jejunum, ganglia length (Figure 8D) and area (Figure 8F) were significantly greater in the Mallard compared to the Tufted Duck and the Green-Winged Teal (*p* < 0.05). For both parameters, no significant differences were observed between the Tufted Duck and the Green-Winged Teal. Ganglia width (Figure 8E) did not differ significantly among the examined species.

In the ileum, ganglia width (Figure 8H) was greatest in the Tufted Duck, showing significant differences compared to the Mallard (*p* < 0.001) and the Green-Winged Teal (*p* < 0.05), with no significant differences between the Mallard and the Green-Winged Teal. Ganglia area (Figure 8I) was also largest in the Tufted Duck compared to the other species (*p* < 0.05), with no significant differences observed between the Mallard and the Green-Winged Teal. Ganglia length (Figure 8G) in the ileum did not differ significantly among the examined species.

In the cecum, ganglia length (Figure 8J) was lowest in the Tufted Duck and significantly smaller compared to the Mallard (*p* < 0.01) and the Green-Winged Teal (*p* < 0.05); no significant differences were observed between the Mallard and the Green-Winged Teal. Ganglia width (Figure 8K) was lowest in the Mallard compared to both the Tufted Duck and the Green-Winged Teal (*p* < 0.05), with no significant differences between the latter two species. No significant differences in ganglia area were observed among the examined species (Figure 8L).

## 4. Discussion

Our study, comparing the morphometry of the GIT in three species of ducks, Mallard (*Anas platyrhynchos*), Tufted Duck (*Aythya fuligula*), and Green-Winged Teal (*Anas crecca*, revealed distinct differences in gastrointestinal structure that may be related to the diet and feeding strategies of these birds. However, it should be noted that some structural dimensions of the GIT, especially those related to the overall size of specific intestinal segments, may be partially dependent on the general body size of the species. Although our findings primarily reflect adaptations associated with diet and habitat use, it is possible that some of the structural differences in the GIT are also influenced by allometric relationships linked to overall body size. Including body mass measurements in future comparative studies would allow for a more precise separation of size-related effects from those driven by ecological and dietary factors. In addition to dietary and ecological factors, some of the interspecific differences observed may be related to phylogenetic constraints. In our study, the Tufted Duck consistently exhibited thicker longitudinal muscle layers in the duodenum, ileum, and cecum, as well as larger enteric ganglia in the duodenum and ileum, compared to the Mallard and the Green-Winged Teal. These traits occurred despite marked differences in foraging strategies within the genus *Anas*, suggesting that certain structural parameters of the GIT may be conserved within phylogenetic lineages. This pattern is consistent with the findings of Duque-Correa et al., who, in an analysis of 390 bird species, demonstrated a strong phylogenetic signal in small intestine length and cecum size, independent of diet and body mass [7]. Similarly, Hunt et al., in their comparative study of 155 avian species, confirmed that cecal length remains significantly correlated with phylogenetic relatedness even after accounting for body mass and dietary category [16]. Nevertheless, our findings suggest that parameters such as villus length or muscle layer thickness are influenced primarily by dietary and ecological factors, rather than by general intestinal size or body mass. Our findings demonstrated, among others, thicker muscle layers in the Tufted Duck (diet with hard-textured food), longer and wider villi in the Mallard (omnivorous diet rich in plant material and fiber), and shorter and narrower villi in the Green-Winged Teal (highly digestible diet).

Among the studied duck species, the Tufted Duck exhibited the thickest muscular layer in the duodenum and ileum, particularly in the longitudinal layer. This morphological feature may reflect the species’ ecological specialization as a diving forager that consumes hard, protein-rich, mechanically resistant food such as mollusks, crustaceans, and insect larvae, in contrast to dabbling ducks, which primarily ingest soft aquatic vegetation near the water surface [17]. The duodenum, as the initial segment of the small intestine, plays a central role in receiving chyme from the gizzard and initiating enzymatic digestion. In Tufted Ducks, the digesta entering the duodenum is often denser and more heterogeneous, requiring stronger peristaltic contractions for effective mixing and forward propulsion [18]. A thicker muscularis externa in this region may therefore enhance the ability to homogenize chyme and regulate its transit rate, ensuring sufficient contact time with digestive enzymes and optimal nutrient extraction [19]. Similar relationships such as stronger intestinal musculature in species with animal-based or hard diets, have been previously reported in other diving birds [20]. Interestingly, similar thickening of intestinal muscle layers has been described in domestic Pekin ducks fed diets high in plant-derived fiber where increased bulk and viscosity of digesta stimulated muscular hypertrophy [21]. Although the dietary source of mechanical challenge differs, the morphological outcome illustrates a common adaptive principle; the intestinal musculature responds to the physical complexity of ingested material.

The avian GIT demonstrates considerable flexibility, with its morphology capable of adjusting to both developmental needs and dietary challenges. In domestic birds such as broilers, turkeys, and Japanese quails, numerous studies have demonstrated that diets rich in structural components, e.g., whole grains, wheat fiber, or high-fiber feeds, lead to significant changes in gastrointestinal morphology [22,23,24]. For instance, Hiżewska et al. [17] showed that in domestic geese, the thickness of the intestinal wall progressively increases during post-hatching development. These changes reflect the natural maturation of the digestive system as it becomes functionally adapted to process increasing volumes and complexity of ingested material. A similarly structured example is observed in laying hens, where the intestine develops a distinct four-layered muscularis externa that facilitates vigorous propulsion of digesta [25]. While these ontogenetic modifications highlight the inherent capacity of the avian intestine to undergo structural remodeling, they occur under controlled dietary conditions. While informative, such examples from domestic birds are not always directly comparable to wild species, which face fluctuating environmental pressures and must physiologically respond to variable and often mechanically demanding diets. For example, Ricklefs found that among passerine birds, species consuming more seeds or fruits, exhibited thicker intestinal muscle and mucosal layers and longer villi, compared to primarily insectivorous birds, which is comparable to our results [26]. Conversely, Kleyheeg et al. [27] showed that in Mallards, adaptation to different diets influenced digestive performance, particularly seed retention time and digestion efficiency, even in the absence of measurable changes in digestive organ size. This suggests that functional digestive adaptation in wild birds may be achieved not only through structural remodeling, but also via subtle physiological adjustments such as altered enzyme activity, retention dynamics, or pH regulation [27].

In the Mallard, whose omnivorous diet consists mainly of marsh plant and soft parts of macrophytes [28], longer villi and greater crypt depth were observed in the duodenum and jejunum compared to the other species. This is a typical response to a fiber-rich diet, which requires a larger absorptive and secretory surface [29]. Comparable findings have been reported in domestic poultry by Rezaei et al. who observed that an increase in dietary fiber content in quails led to an increase in the mass of intestinal segments and significant elongation and thickening of intestinal villi [30]. Similarly, Novotný et al. also demonstrated that broilers fed a diet with coarser particle fractions developed significantly elongated intestinal villi and thickened mucosal layers, indicating an adaptive response aimed at enhancing the absorptive surface area under increased mechanical and digestive load. In addition to these intestinal changes, the same diet also resulted in increased gizzard mass, reflecting a broader gastrointestinal adaptation to the physical characteristics of the feed [22]. For contrast, the Green-Winged Teal, which prefers an insectivorous diet rich in small aquatic organisms and seeds [31], exhibited shorter but more numerous villi per 1 mm in the duodenum and jejunum, and thinner muscle layers, which is typical for a diet with high digestibility and lower structural fiber content [32]. Furthermore, the Mallard exhibited the deepest crypts in the caecum. In this part of the intestine, goblet cells play a crucial role in producing mucus, which is essential for protecting the epithelium, regulating the local microenvironment, and maintaining appropriate conditions for microbial fermentation [33]. Deeper crypts may indicate an increased number or activity of these cells, potentially representing an adaptive response to the prolonged intake of a fiber-rich diet. Such a diet, which demands enhanced protective and regulatory mechanisms, may stimulate the development of structures that support adequate mucin production and secretion [34].

Seasonal variation in food availability can significantly modulate the morphology of the GIT, and our July results illustrate this well. In summer, the Tufted Duck, whose diet includes numerous mussels, crustaceans, and benthic insect larvae, exhibited a thicker muscular layer and larger intestinal ganglia—typical adaptations to mechanically demanding food [31]. The Mallard, feeding in summer on aquatic plants and fiber-rich grains, had longer and deeper villi and crypts, consistent with experiments showing that a high-fiber diet in mallards increases the absorptive surface area of the intestine [35]. The Green-Winged Teal, feeding in the same season mainly on small aquatic organisms and insects, had thinner intestinal walls and narrower villi and crypts, which corresponds to the lower mechanical demands of digestion [4]. The diet of waterfowl undergoes significant changes in winter due to the limited availability of fresh aquatic and terrestrial vegetation and the increased availability of seeds and grains from agricultural fields or wetland habitats. For example, in the study by Delnicki and Reinecke, it was shown that during winter mallards markedly increase the proportion of seeds and grains in their diet, which then account for over 90% of their total food intake [36]. Although our data cover only the summer season, the clear correlation between morphological differences and seasonal diet suggests that comparative studies across different seasons could reveal the extent of adaptive, seasonal plasticity of the GIT in these species.

Beyond physiological plasticity and microanatomical remodeling, additional support for diet- and habitat-driven gastrointestinal adaptation comes from broader ecological and morphological studies. For instance, a study by Jónsson et al. showed that gastrointestinal morphology, especially gizzard and cecum length, varies depending on habitat and diet composition, interpreted as an adaptation to food with different fiber content. Larger digestive organs were observed in individuals from marshy habitats, where the diet was richer in fiber, whereas larger leg muscles occurred in individuals from habitats requiring greater mobility. Notably, variability in organ size was already evident in juvenile birds, indicating early development of adaptive traits. These findings support our interpretation of morphological differences in the intestines of the three duck species as a potential reflection of divergent feeding and habitat strategies [37].

Morphometric analysis of enteric ganglia revealed clear interspecies differences across intestinal segments, paralleling variations in intestinal wall structure. In the duodenum, the Tufted Duck had the largest ganglia, in terms of length, width, and area, coinciding with its particularly thick muscularis externa in this region. Based on available data, this correspondence suggests that an enhanced ENS network may be required to coordinate stronger peristaltic contractions in response to denser, mechanically demanding chyme typical of this species’ diet [38,39]. In the jejunum, the longest and largest ganglia were observed in the Mallard. Longer ganglia in this segment may indicate advanced mechanisms for controlling prolonged peristalsis, potentially related to longer digesta retention time in this species. In the cecum of Mallards, ganglia were longer but narrower, which may facilitate synchronous motor waves along the entire cecal length, promoting fermentation [40]. In contrast, Tufted Ducks and Green-Winged Teals had wider but shorter ganglia in the cecum—this pattern suggests a more localized branching of neural plexuses, facilitating faster emptying of this gut section [41].

Observed interspecies differences in ENS morphology may reflect not only genetically determined developmental patterns but also the innate plasticity of this system. ENS plasticity may involve both neurogenesis—the formation of new enteric neurons—and synaptic remodeling, consisting of structural and functional modifications of existing neural circuits. These mechanisms have been described in vertebrates in response to changes in diet composition, food load, or intestinal motility requirements [42]. In our study, particularly interesting in this context were the very large ganglia dimensions in the duodenum and ileum of the tufted duck, as well as the increased surface area and length of ganglia in the jejunum of the mallard. Enlargement of neuronal structures may result, among others, from an increased number of neurons (neurogenesis), expansion of neuronal processes, reorganization of synaptic connections, or an increase in the volume of glial cells. Although our morphometric measurements do not allow us to clearly indicate which of these mechanisms predominates, the literature suggests that both neurogenesis and synaptic remodeling can occur in the adult ENS in response to environmental stimuli and dietary changes [43,44]. Such processes could explain some of the observed morphological variability, indicating that the ENS of wild waterfowl retains the ability for structural adaptation even in adulthood.

Although the literature on ENS morphology in waterfowl remains limited, available data from poultry research indicate high adaptability of this system [45]. Our findings, showing significant differences in intestinal wall and ganglia morphometry among the three wild duck species, point to their distinct trophic strategies and gastrointestinal adaptation to diet type. In the context of literature data on broilers, where replacing soybean protein with chickpea protein triggered reorganization of neural plexuses and alterations in intestinal structure, our observations may also suggest that, in wild ducks, the ENS and intestinal morphology undergo adaptive modifications in response to food quality and foraging conditions [46,47].

## 5. Conclusions

This study confirms that both the morphological structure of the GIT and the organization of the enteric nervous system are strongly influenced by the dietary preferences and ecological strategies of duck species. The Tufted Duck, which feeds on hard aquatic organisms, exhibited thicker muscular layers and larger enteric ganglia, supporting enhanced peristalsis. The Mallard, with its omnivorous diet, showed villi and crypt morphology adapted for efficient nutrient absorption. In contrast, the Green-Winged Teal, which consumes easily digestible, insect-based food, displayed simplified intestinal morphology and thinner muscle layers. These results indicate that both morphometric and neuroregulatory features of the digestive system undergo adaptive modifications in response to environmental pressures and dietary composition. Such adaptations may directly translate into digestive efficiency—through the optimization of mechanical breakdown and transport of ingesta, the increase in the intestinal absorptive surface area, or the reduction in the energetic costs of digestion—as well as into the ability to effectively exploit specific habitats and food resources. The findings contribute to a better understanding of the functional and neuroanatomical adaptations of waterfowl to their trophic niches. Moreover, the applied morphometric and immunohistochemical methods proved useful in detecting fine-scale anatomical differences between species. This study provides a valuable reference point for future ecological, comparative, and evolutionary research on avian digestive systems.

## Figures and Tables

**Table 1:** Characteristics of antibodies used for immunohistochemical staining.

Target Antigen	Antibody Type	Host Species	Manufacturer	Catalog Number	Dilution/Format
Anti-Human Neuronal Protein HuC/HuD (anti-HuC/D)	Monoclonal, primary	Mouse	Thermo Fisher Scientific	A-21271	1:200
Anti-mouse/rabbit (secondary)	Polyclonal, HRP-conjugated secondary	Goat	ImmunoLogic	DPVB-HRP	RTU ^1^

^1^ RTU—ready to use.

## Data Availability

The raw data supporting the conclusions of this manuscript will be available upon request.

## Abbreviations

The following abbreviations are used in this manuscript:

ANOVA	Analysis Of Variance
DAB	3,3′-Diaminobenzidine
ENS	Enteric Nervous System
GIT	Gastrointestinal Tract
HRP	Horseradish Peroxidase
IHC	Immunohistochemistry
PBS	Phosphate-Buffered Saline
RTU	Ready To Use
SEM	Standard Error of the Mean
